# Silver Nanoparticles at Biocompatible Dosage Synergistically Increases Bacterial Susceptibility to Antibiotics

**DOI:** 10.3389/fmicb.2020.01074

**Published:** 2020-05-27

**Authors:** Deepak S. Ipe, P. T. Sudheesh Kumar, Robert M. Love, Stephen M. Hamlet

**Affiliations:** ^1^School of Dentistry and Oral Health, Griffith University, Gold Coast, QLD, Australia; ^2^Menzies Health Institute Queensland, Griffith University, Gold Coast, QLD, Australia

**Keywords:** silver nanoparticles, antimicrobial, resistance, synergistic, antibiotics, susceptibility

## Abstract

Antibiotics used to treat bacterial infections can become ineffective over time or result in the emergence of antibiotic resistant pathogens. With the advent of nanotechnology, silver nanoparticles (AgNPs) have gained significant attention as a therapeutic agent due to the well-known antimicrobial properties of silver. However, there are concerns and limited literature on the potential cytotoxicity of nanoparticles at effective antimicrobial concentrations. AgNPs prepared from silver nitrate with glucose reduction were characterized by surface plasmon resonance, dynamic light scattering, zeta potential analysis and transmission electron microscopy. The cytotoxicity of AgNPs towards human gingival fibroblasts over 7 days was determined using cell proliferation assays and confocal microscopy. AgNP MIC and antibacterial effects alone and in combination with 11 antibiotics were determined against a panel of nine microbial species including gram-positive and gram-negative bacterial species. AgNPs concentrations ≤ 1 μg/mL were non-cytotoxic but also showed no antibacterial effects. However, when combined with each of eleven antibiotics, the biocompatible concentration of AgNPs (1 μg/mL) resulted in significant inhibition of bacterial growth for multiple bacterial species that were resistant to either the antibiotics or AgNPs alone. This study presents a promising strategy with further testing *in vivo*, to develop novel antimicrobial agents and strategies to confront emerging antimicrobial resistance.

## Introduction

The emergence of multidrug-resistant bacterial strains is making the continued use of antibiotics increasingly ineffective. Moreover, failure to eliminate bacteria at the planktonic stage allowing the subsequent formation of a bacterial biofilm, increases the resistance of bacteria to both the host immune system and antibiotics (Croes et al., 2018). Silver is a well-known antimicrobial agent that has been used clinically, well before the discovery of penicillin and numerous commercially available products including wound dressings, creams, and coatings utilize silver for its anti-bacterial effects (Abbasi et al., 2016; Rafique et al., 2017; Bapat et al., 2018).

Silver ions, however, are relatively reactive and can form insoluble precipitates *in vivo* such as AgCl or can encounter significant reduction in their antibacterial efficacy due to interactions with blood proteins like albumin. Interestingly, silver nanoparticles (AgNPs) on the other hand are comparatively less reactive than silver ions and may potentially be more suited for use in clinical and therapeutic applications (Chen and Schluesener, 2008; Madhumathi et al., 2010).

Demonstrated to be effective in killing both gram-negative and gram-positive bacteria (Taglietti et al., 2012), the antibacterial properties of AgNPs are related to both the small size of the particles and their propensity to form silver ions (Boudreau et al., 2016). The antibacterial properties also tend to increase with decreasing particle size due to the increased surface area to mass ratio and higher surface reaction activities (Lok et al., 2007). AgNPs have a multi-level mode of action influencing many bacterial structures and metabolic processes including inactivating bacterial enzymes (Li et al., 2011), disrupting cell wall, metabolic processes (Cui et al., 2013) and increasing cell permeability (Morones et al., 2005; Saravanan et al., 2018). AgNPs can also interact with DNA (Li et al., 2011) or generate reactive oxygen species (Xu et al., 2012) which damage biomacromolecules (Cabiscol et al., 2000).

These actions of AgNPs have also been shown to enhance, either additively or synergistically, the antibacterial effects of antibiotics against multiple bacterial species including antibiotic resistant strains (Ruden et al., 2009; Fayaz et al., 2010; Hwang et al., 2012; McShan et al., 2015; Deng et al., 2016; Panacek et al., 2016) and more recently, synergistic antifungal effects with antifungal agents (Patra and Baek, 2017). Such synergistic actions can significantly reduce the need for high antibiotic dosages and therefore minimize side effects.

As AgNPs are among the most widely used nanomaterial in consumer products, increased use in the food industry has led to public concern regarding their safety and toxicity with long-term use (Chen and Schluesener, 2008; Wijnhoven et al., 2009). Unfortunately, AgNPs can act like a double-edged sword, i.e., they can eliminate bacteria but have also been shown to induce cellular cytotoxicity; *in vitro* cell culture studies have shown toxic effects of AgNPs in a number of human cell lines (Shi et al., 2018; Botha et al., 2019; Liao et al., 2019). Similarly, *in vivo* animal studies in rodents have also shown toxic effects of AgNPs due to their accumulation in the liver, spleen, and lung (Alessandrini et al., 2017; Vidanapathirana et al., 2018).

AgNP-mediated cytotoxic effects in mammalian cells depend greatly on the physico-chemical properties of the nanoparticles including their size, shape, surface charge, oxidation state and agglomeration condition as well as the dosage and cell type encountered (Dakal et al., 2016). These physico-chemical properties are in turn dependent upon the nanoparticle synthesis method. By optimizing the synthesis procedures and using appropriate reducing agents and stabilizers, ‘tuneable’ control over the shape, size, and charge distribution of the AgNPs can be achieved (El-Rab, 2012) to enhance the biocompatibility and bioactivity of AgNPs for various nanotechnological applications.

A majority of the previous studies which examined the bacterial killing of AgNPs and the synergistic effect of antibiotics, did not assess any potential cytotoxicity of the AgNPs (Fayaz et al., 2010; Hwang et al., 2012; Deng et al., 2016; Patra and Baek, 2017). If cytotoxicity was assessed, the studies were usually short-term (<5 days), (Panacek et al., 2016; Punjabi et al., 2018) in duration unable to show longer-term cytotoxic effects. Robust data on enhancing antibiotic potential in conjunction with biocompatible doses of AgNPs (Botha et al., 2019) is therefore lacking.

Therefore, the aims of this study were to (1) characterize AgNPs produced using a non-toxic silver reduction methodology and assess their biocompatibility with human fibroblasts over 7 days. (2) Based on the cytotoxicity data, using a biocompatible concentration of AgNPs, assess the antibacterial effects of the AgNPs against multiple bacterial species, including both gram-negative and gram-positive bacteria. (3) Evaluate the potential synergistic effects of AgNPs when used in combination with a wide spectrum of antibiotics.

## Materials and Methods

### Synthesis of AgNPs

Soluble starch (0.20% wt/vol) was dissolved in boiling water and stirred until complete dissolution. To this solution, 0.1M silver nitrate (AgNO_3_) was added and mixed to a 1/60 dilution. Following complete dissolution, 0.1M D-glucose was added drop wise to achieve 1/40 dilution and then maintained at 60°C for 2–3 h. A drop of 1M sodium hydroxide (NaOH) solution was added turning the solution light yellow indicating the formation of AgNPs (Srinivasan et al., 2013). All chemicals with ≥99% (where possible) purity, were purchased from Sigma-Aldrich, Australia.

### Characterization of AgNPs

#### Surface Plasmon Resonance

Confirmation of AgNP formation was demonstrated by surface plasmon resonance (SPR) obtained from the absorbance spectrum recorded between 300 and 600 nm at 2 nm intervals (POLARstar Omega plate reader, BMG Labtech). Inductively Coupled Plasma Optical Emission Spectroscopy (ICPOES) was subsequently used to determine the concentration of AgNPs (Perkin Elmer Optima 8300 dual view ICPOES). The ICPOES dual monochromators for UV and visible range emission have a wavelength range from 165 to 800 nm. Emitted wavelengths were measured using a sealed charged couple device detector.

#### Transmission Electron Microscopy (TEM) Imaging

To examine the AgNPs morphological features including size, TEM was performed. One drop of 52 μg/mL AgNPs was placed onto a carbon coated copper grid and allowed to settle for 5 min then allowed to air dry at room temperature. TEM was performed at 100 kV (Hitachi HT7700).

#### Dynamic Light Scattering (DLS) and Zeta Potential

DLS and Zeta potential of prepared AgNPs was measured following previously published protocols (Clogston and Patri, 2011) at a scattering angle of 173° and a temperature of 25°C (Malvern Zetasizer Nano ZS, Malvern Instruments, Malvern, United Kingdom) to confirm size and charge of AgNPs. Sample solution was diluted (1/10) in water and the following parameters used for subsequent calculations: dispersant – water; viscosity – 0.8872 cP; refractive index – 1.33; dielectric constant – 78.5.

### Biocompatibility of AgNPs

Human gingival fibroblasts were isolated from extracted healthy, but impacted or unwanted tooth as reported previously (Somerman et al., 1988; Ivanovski et al., 2001). For this study gingival fibroblast cells named MD-HGF, passage number 5, were used and ethical approval for the use of the redundant cells/tissues was attained through the Griffith University Human Research Ethics committee (DOH/17/7/HREC). Cells were cultured in Dulbecco’s Modified Eagle’s Medium (DMEM) cell culture media supplemented with 5% Fetal Bovine Serum (FBS), 1X Minimum Essential Medium (MEM) non-essential amino acids and 1% Penicillin-Streptomycin. This supplemented DMEM is referred as complete DMEM (cDMEM). Cells were cultured in 75 m^2^ flasks at 37°C in a 5% CO_2_ atmosphere until 80–90% confluent before passaging. All cell culture reagents were purchased from Thermo Fisher Scientific, United States.

Fibroblasts (5 × 10^4^ cells/well) were seeded in triplicate into 24 well plates with 1.5 mL/well cDMEM and cultured with AgNPs (0.5, 1.0, 1.5, and 2.0 μg/mL) in cDMEM. Cell viability was subsequently assessed at days 1, 3, 5, and 7 of culture using 0.4% trypan blue (Sigma-Aldrich, Australia) cell counting (Strober, 2015) and a CCK-8 viability assay (Auspep, Australia). The percentage viability of treated wells was calculated by comparing with untreated control wells.

To visualize any morphological changes in response to the different concentrations of AgNPs including cell death, a live and dead assay was performed and visualized under confocal microscopy. For this, the cells were incubated at room temperature for 30 min with 1 μg/mL of Fluorescein Diacetate (FDA, Thermo Fisher Scientific, Australia) to visualize live cells (green) and 2 μg/mL of Propidium Iodide (PI, Thermo Fisher Scientific, Australia) to observe dead cells (red). The samples were then imaged at room temperature using a Nikon eclipse Ti confocal Microscope at Ex/Em 488/526 nm for FDA and Ex/Em 493/636 nm for PI at 10X magnification.

### Antibacterial Effects

#### Bacterial Species

All bacterial species used in this study were obtained from the American Type Culture Collection (ATCC) via a local distributer (In Vitro Technologies Inc., Australia). Gram-positive bacterial species included: *Staphylococcus aureus* ATCC 25923, Methicillin Resistant *Staphylococcus aureus* (MRSA) ATCC 4330, *Streptococcus mutans* ATCC 25175, *Streptococcus oralis* ATCC 35037, *Streptococcus gordonii* ATCC 49818, and *Enterococcus faecalis* 700802. Gram-negative bacterial species included *Escherichia coli* ATCC 25922, *Aggregatibacter actinomycetemcomitans* ATCC 33384 and *Pseudomonas aeruginosa* ATCC 27853. Fresh overnight cultures of gram-positive and gram-negative bacterial species were grown in Brain Heart Infusion (BHI, Thermo Fisher Scientific, Australia) broth/agar and in lysogeny broth/agar (LB, Thermo Fisher Scientific, Australia) respectively unless otherwise stated.

#### Antimicrobial Effects of AgNPs

Minimum inhibitory concentration (MIC) of AgNPs was assessed using a broth microdilution method. Two fold serial dilutions of AgNPs (1–40 μg/mL) that were inoculated with approximately 1 × 10^4^ Colony Forming Unit (CFU) of bacteria from an overnight culture, and grown in 200 μl of total volume of either BHI or LB, were incubated at 37°C in 96-well microtiter plates for up to 24 h. Optical density at 600 nm were recorded at 0, 6, 12, and 24 h (POLARstar Omega plate reader, BMG Labtech).

To assess the antibacterial effect of (a) antibiotics (b) AgNPs and (c) antibiotics combined with biocompatible dose of AgNPs (1 μg/mL), the disk diffusion method (Kirby-Bauer) was followed according to the Clinical and Laboratory Standards Institute (CLSI) guidelines (Hwang et al., 2012; Punjabi et al., 2018; Weinstein, 2018). The antibiotics (Table 1 and Supplementary Figures S1A,B) were tested at their standard disk potency concentrations on BHI or LB agar (Weinstein, 2018).

**TABLE 1:** Details of the antibiotics used and bacterial species tested.

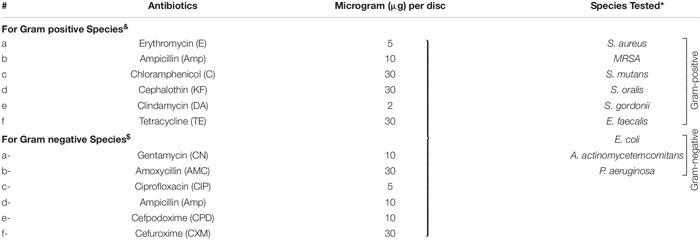

Each species was tested against all the antibiotics listed both with and without 1 μg AgNPs (*). Lower case letters correspond to the antibiotic tested shown in Supplementary Figures S1A,B (#). Antibiotics generally accepted to use against (&) gram-positive and ($) gram-negative bacterial species.

Fresh overnight microbial culture (100 μl), adjusted to approximately 2 × 10^8^ CFU/mL, was evenly spread onto the agar. To assess the effect of AgNPs on bacterial growth using the disk diffusion method, 1, 2, and 5 μg/mL of AgNPs were impregnated into 6 mm circular Whatman filter disks and placed onto agar plate containing bacterial spread. For the studies of antibiotics alone (Table 1) and antibiotics combined with 1 μg of AgNPs (i.e., biocompatible concentration), ready-to-dispense pre-loaded antibiotics disks (6 mm, Oxoid, Thermo Fisher Scientific, Australia) with or without AgNPs were placed onto the agar plate containing the bacterial spread. Plates were then incubated at 37°C overnight and the diameter (including disk) of the Zone of Inhibition (ZoI) for AgNPs, antibiotics and AgNPs in combination with antibiotics against each bacterial species were then recorded in millimeters.

### Statistical Analysis

Cell viability and antibacterial effects of AgNPs with or without antibiotics were analyzed using a Two-way ANOVA with post hoc analysis (Tukey’s multiple comparisons test). All statistical analyses were carried out using GraphPad Prism software version 8.0.1. A *p*-value of <0.05 was considered significant.

## Results

### Synthesis and Characterization of AgNPs

**FIGURE 1:**
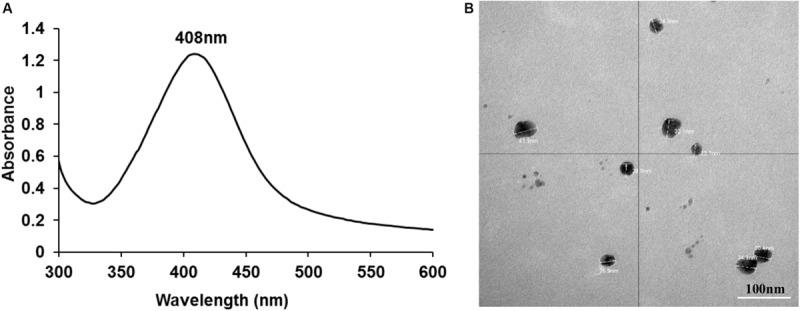
UV-visible spectroscopy demonstrating a SPR band with a peak at 408 nm **(A)** and TEM image showing individual nano particles of silver with an average size of ∼26 nm **(B)**.

AgNPs were successfully synthesized using AgNO_3_ and D-glucose as the reducing agent. A pale-yellow color of the final solution indicated the formation of AgNPs. Characterization using UV-visible spectroscopy demonstrated a SPR band with a single peak at 408 nm (Figure 1) confirming the presence of AgNPs.

ICPOES indicated that the concentration of the prepared AgNPs was 52 μg/mL. TEM analysis demonstrated spherical shaped AgNPs with an average size of ∼26 nm which were not aggregated. Particle sizes of ∼26 nm obtained from the DLS analysis was consistent with TEM analysis. The charge of synthesized nanoparticles determined by Zeta potential was −8.9 mV suggesting incipient instability.

### Biocompatibility of AgNPs

**FIGURE 2:**
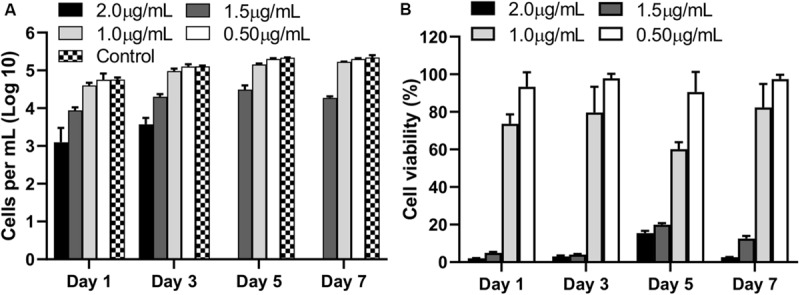
Viable cell counts **(A)**, and percentage viability **(B)**, compared to untreated control cells (no AgNPs) at each observation time points over 7 days following culture with different concentrations of AgNPs. Data are representative of at least three independent assays and show mean values ± standard errors of the means.

The biocompatibility of AgNPs assessed by quantifying the numbers of viable cells and assessing the percentage of metabolically active cells (CCK-8) revealed 2 μg/mL or higher concentration of AgNPs was toxic to cells (*p* < 0.0001) with no viable cells by Day 5 compared to untreated control cells (Figure 2A). At 1.5 μg/mL of AgNPs, some cells survived albeit at a low density (*p* < 0.0001) compared to control. Interestingly, at a concentration of 1 μg/mL or lower no cytotoxicity was observed compared to untreated controls, in fact 1 μg/mL and 0.5 μg/mL of AgNPs showed no significant toxicity compared to the 2 μg/mL at all time points tested over 7 days (Figure 2A; *p* < 0.05).

The results of the cell metabolic assay represented as percentage viability against untreated cells, showed a similar trend where 2 μg/mL showed significantly strong cytotoxic effects when compared to untreated wells (*p* < 0.0001) at all time points. No significant cytotoxicity was observed at 1 μg/mL or lower concentrations of AgNPs (Figure 2B).

**FIGURE 3:**
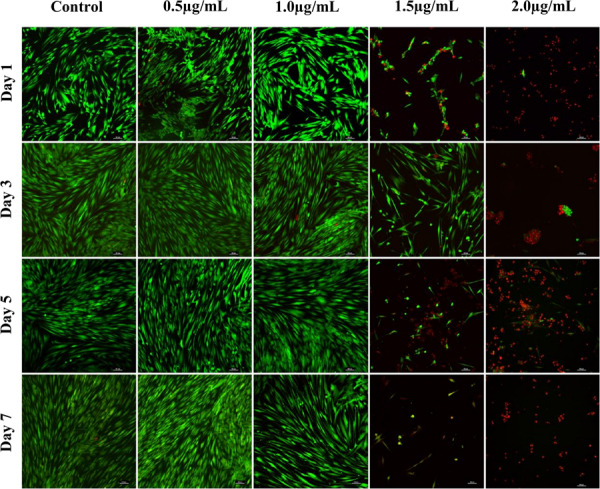
Confocal microscopy images of live (green) and dead (red) fibroblast cells treated with different concentrations of AgNPs (0.5, 1.0, 1.5, and 2.0 μg/mL) for 7 days. Scale bars used were at 100 μm.

Confocal images of live and dead cells (Figure 3) were consistent with the results of both biocompatibility assays (Figure 2). Concentrations of AgNPs ≥ 1.5 μg/mL clearly showed significant cell death over the 7-day period (Figure 3) whereas culture with 1 μg/mL or lower concentrations of AgNPs demonstrated cells were live and healthy, similar to untreated control samples.

### Antibacterial Potential

#### AgNPs Alone

**FIGURE 4:**
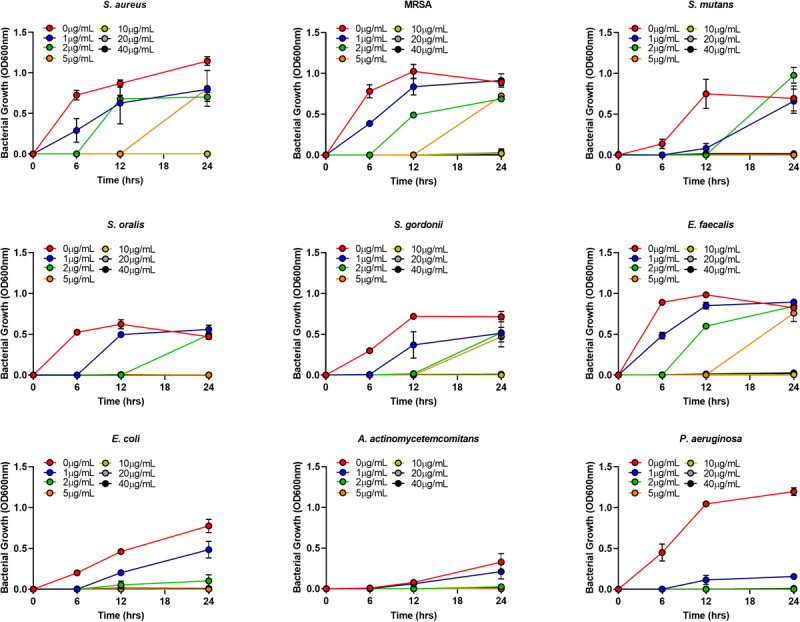
Gram-positive and gram-negative bacterial species growth in BHI/LB at different concentrations (0, 1, 2, 5, 10, 20, 40 μg/mL) of AgNPs.

**FIGURE 5:**
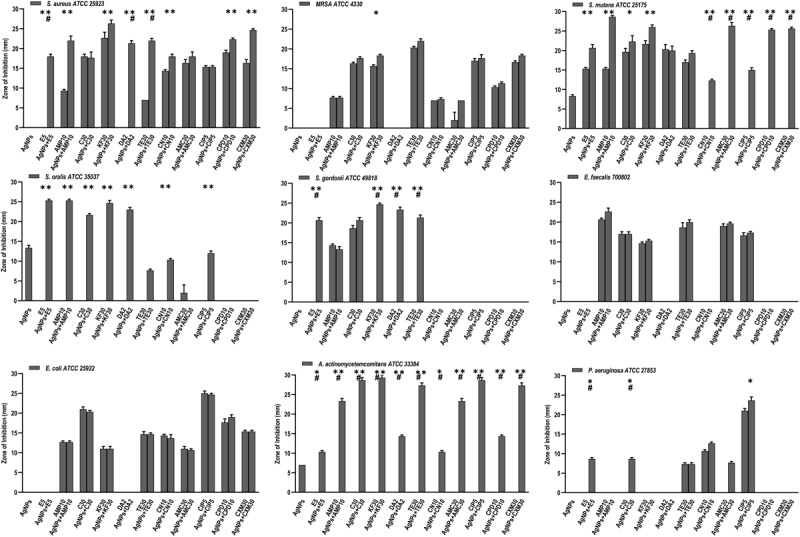
Antibacterial activity of AgNPs (1 μg), antibiotics, and AgNPs (1 μg) combined with each of the 11 antibiotics, against 9 bacterial species. Significance levels (*p < 0.05, **p < 0.0001, ^#^*p* < 0.05) indicate increases in ZoI of combined AgNPs and antibiotic compared to either antibiotic (*) or AgNPs alone (#).

The MIC of AgNPs assessed using the broth microdilution method showed none of the bacteria tested had growth inhibition at the biocompatible concentration of 1 μg/mL (Figure 4 and Table 2). The MIC of AgNPs against gram-positive bacterial species (5–10 μg/mL) were generally higher than that for gram-negative species. *A. actinomycetemcomitans* and *P. aeruginosa* showed susceptibility at 2 μg/mL although *E. coli* was more resistant with an MIC of 5 μg/mL (Figure 4 and Table 2). Also using the disk diffusion method, almost all the bacterial species tested did not demonstrate any ZoI with the maximum possible biocompatible dose of AgNPs, i.e., 1 μg/mL (Figure 5 and Supplementary Figures S1A,B). Overall, both broth microdilution and disk diffusion methods were consistent in demonstrating AgNPs at 1 μg/mL had no significant antibacterial effects (Figures 4, 5).

**TABLE 2:** Minimum inhibitory concentration (MIC) of AgNPs for both gram-positive and gram-negative bacterial species tested.

***#***	**Bacterial species tested**	**MIC of AgNPs (μg/mL)**
**Gram positive bacterial Spp.**		
**1**	*S. aureus*	10
**2**	MRSA	10
**3**	*S. mutans*	5
**4**	*S. oralis*	5
**5**	*S. gordonii*	10
**6**	*E. faecalis*	10
**Gram negative bacterial Spp.**		
**7**	*E. coli*	5
**8**	*A. actinomycetemcomitans*	2
**9**	*P. aeruginosa*	2

#### Antibiotics Alone

Of the eleven antibiotics tested (Table 1), none of the gram-positive bacterial species demonstrated susceptibility to more than three antibiotics (CLSI) (Weinstein, 2018) despite some of these antibiotics are generally accepted to treat gram positive bacterial infections, i.e., Erythromycin (E5), Chloramphenicol (C30), Clindamycin (DA2), and Tetracycline (TE30) (Figure 5). Of the gram-negative bacterial species tested, *P. aeruginosa* and *A. actinomycetemcomitans* were resistant to almost all eleven antibiotics except for *P. aeruginosa* susceptibility to Ciprofloxacin (CIP5) (Figure 5). *E. coli* showed susceptibility to C30, TE30 and CIP5 with an ‘intermediate’ MIC towards Gentamycin (CN10) and Cefuroxime (CXM30) (Figure 5).

#### AgNPs and Antibiotics Combined

The synergistic effects of the biocompatible dose of AgNPs when combined with each of the eleven antibiotics was tested against all the bacterial species. Many significant synergistic antibacterial effects were demonstrated by the AgNP and antibiotic combinations (Figure 5). For example, *S. aureus* was resistant to both AgNPs alone, gram-positive targeting antibiotics – E5, DA2, TE30 and other tested antibiotics Amp10, CPD10, CXM30 (*p* < 0.0001). However, in combination with 1 μg/mL of AgNPs, the antibacterial effectiveness of each of these antibiotics increased synergistically from no growth inhibition (i.e., resistant) into the susceptible range (Figure 5, CLSI) (Weinstein, 2018). Interestingly, where ‘intermediate’ antibiotic sensitivity was demonstrated, e.g., with C30 for MRSA, *S. mutans, S. gordonii*, the addition of AgNPs enhanced the antimicrobial effectiveness to ‘susceptible’ (Figure 5). This additive effect of the AgNPs is also demonstrated in combination with the antibiotics KF30, CN10, CPD10, and CXM30 with both gram-positive and gram-negative bacterial species. With MRSA, no large synergistic effects (*p* < 0.01) were observed, rather only small additive effects to the existing antibiotic effect, e.g., C30 and KF30, enhancing the antimicrobial efficiency of these antibiotics from ‘intermediate’ to ‘susceptible’ (Figure 5 and Supplementary Figures S1A,B).

Similarly, if using antibiotics alone, *S. mutans* (E5, Amp10, C30, KF30, TE30), and *S. gordonii* (C30) are deemed ‘resistant’ or ‘intermediate’. The addition of AgNPs, however, increases the sensitivity towards susceptible. Significantly, however, if the antibiotic was ineffective in inhibiting the bacterial growth, e.g., with all the antibiotics tested against *S. oralis* and *A. actinomycetemcomitans*, the addition of AgNPs both synergistically and significantly increased the ZoI with 7 of the 11 antibiotics for *S. oralis* and 10 of the 11 antibiotics for *A. actinomycetemcomitans* (of which >5 of the antibiotics effectiveness increased from resistant to susceptible). This synergistic effect was also shown with *S. gordonii* where significant (*p* < 0.0001) antibacterial effects against E5, KF30, DA2 and TE30 were noted with ZoI’s of >20 mm compared to no effect against the AgNPs or antibiotics alone (Figure 5 and Supplementary Figures S1A,B). Interestingly, antimicrobial effect was not restricted to a class of antibiotics used, i.e., whether antibiotics that target gram-positive or gram-negative bacteria. *S. mutans* for example showed resistance to antibiotics generally used to target gram-negative bacterial species as expected, but when combined with AgNPs, CN10, AMC30, CIP5, CPD10, and CXM30, all showed significant (*p* < 0.0001) inhibition of bacterial growth up to 26 mm ZoI (Figure 5 and Supplementary Figure S1). This trend was also observed for *S. oralis* with AgNPs combined with CN10 or CIP5 (*p* < 0.0001).

## Discussion

This study successfully generated AgNPs by a non-toxic reduction method of silver nitrate using glucose as the reducing agent (Srinivasan et al., 2013). Successful formation of AgNPs was confirmed by UV-visible spectroscopy and TEM followed by DLS and zeta potential analysis. The absorbance spectrum analysis showed a single SPR peak at 408 nm indicating the formation of AgNPs consistent with prior studies (Pal et al., 2007; Srinivasan et al., 2013; Patra and Baek, 2017; Alsammarraie et al., 2018). TEM analysis enabled visualization of the nanoparticle/s physical appearance and approximate size while the charge of the particles was confirmed using DLS and zeta potential, all of which agreed with the reported literature.

The emergence of gram-positive and gram-negative antibiotic resistant pathogens including vancomycin resistant *E. faecalis*, MRSA and *E. coli* are a major threat and burden for healthcare systems worldwide (Boyd et al., 2006; Alvarez et al., 2019; Hwang and Yoon, 2019; Zha et al., 2019). Therefore alternative strategies to antibiotics are urgently required. While the antibacterial potential of AgNPs has been described (Morones et al., 2005; Li et al., 2011; El-Rab, 2012; Taglietti et al., 2012; Polivkova et al., 2017; Saratale et al., 2017; Burduşel et al., 2018; Kshirsagar et al., 2018; Souza et al., 2018; Zheng et al., 2018; Zhu et al., 2018; Some et al., 2019), we are unaware of any studies which have examined the antibacterial potential including antibiotic synergistic effects, of biocompatible concentrations of AgNPs. *In vitro* studies that have assessed the cytotoxicity of AgNPs were either too short, e.g., 24 h or ≤5 days and/or did not consider the antibacterial efficacy (Patra and Baek, 2017; Alsammarraie et al., 2018; Botha et al., 2019; Samuel et al., 2020).

This study clearly demonstrates using a human fibroblast biocompatible concentration of 1 μg/mL, AgNPs in combination with each of eleven commonly used antibiotics, can synergistically increase antimicrobial effects. Moreover, the antibiotics concentrations used in this study are widely accepted as per the CLSI standards. This observation may have a significant impact on the development of novel antimicrobial strategies targeting antibiotic resistant pathogens, as nanoparticles can be easily incorporated into a variety of consumer medical products. Indeed, the Nanotechnology Consumer Products Inventory (CPI) as of 2015 lists 1814 consumer products containing nanomaterials. Perhaps not surprisingly given the history of using silver as an antibacterial agent, AgNPs are the most frequently used nanomaterial accounting for 24% of these products (Vance et al., 2015). Almost one third of these products contain nanomaterials suspended in liquid media intended for dermal exposure. The increasing prevalence of products containing nanomaterials and the level of exposure to such nanomaterials, has in recent times raised public health concerns over potential cytotoxic effects with long term exposure.

For any potential medical application, the absence of any adverse cytotoxic effects from nanomaterials is extremely important, however, AgNPs have indeed been shown to be toxic not only to bacteria and fungi, but also to several animal species and cultured cells (Johnston et al., 2010; Gaiser et al., 2013). A limitation of earlier reported studies assessing nanomaterial cytotoxicity was that the synergistic effects of AgNPs when combined with antibiotics were only observed if the concentrations of the AgNPs and antibiotics reached their own individual MIC. Panacek et al. (2016) however, demonstrated strong synergistic antibacterial effects of AgNPs and antibiotics were possible at concentrations well below the MIC of the individual components, but only included one gram-positive and two gram-negative bacterial species in their study (Panacek et al., 2016). Furthermore, Panacek et al. (2016) showed no significant cytotoxicity towards a murine fibroblast cell line (NIH-3T3) albeit this was only assessed for 24 h.

In the present study we extended these observations to show that at low AgNPs concentrations, i.e., 1.0 μg/mL, the viability of primary human fibroblasts was over 80% even after 7 days of direct culture with the AgNPs. The precise concentration at which AgNPs may be cytotoxic is still unresolved in the literature, primarily due to the wide range of differing methodologies used to produce the nanoparticles and the subsequent *in vitro* testing systems utilized (Pan et al., 2007; Gaiser et al., 2013; Niska et al., 2016; Nakkala et al., 2017; Kshirsagar et al., 2018; Shi et al., 2018). At the biocompatible concentration used in this study, Krajewski et al. (2013) showed AgNPs did not induce any hematologic changes whereas 3 μg/mL significantly increased CD11b expression on granulocytes while 30 μg/mL induced hemolysis of erythrocytes, α-granule secretion in platelets as well as activation of the coagulation and complement cascades (Krajewski et al., 2013).

The biocompatible concentration of AgNPs used in this study was shown to act synergistically with a range of different antibiotics to enhance microbial killing. These results are consistent with previous studies regarding the effects of AgNPs alone and in combination with conventional antibiotics against pathogenic strains (Vijayan et al., 2016; Saratale et al., 2017). Interestingly, synergistic effects were observed with antibiotics targeting both gram-positive and gram-negative bacteria suggesting a non-specific multimodal mechanism of action. Saratale et al. (2017) have suggested that nanoparticles can attach to the bacterial cell membrane forming pits on the cell surface allowing penetration to the cell where AgNPs have a greater affinity to react with sulfur or phosphorous containing biomolecules such as DNA, thereby inhibiting DNA replication and leading to cell death (Singh et al., 2013; Saratale et al., 2017). Similarly, AgNPs may also preferentially attack and disrupt the respiratory chain by interacting with thiol groups present in enzymes such as NADH dehydrogenase (Vijayan et al., 2016). This non-specific antibacterial activity of AgNPs leads to an overall attenuation of the bacteria helping to prevent the development of bacterial resistance.

If, as suggested, AgNPs do not affect bacteria by one specific mode of action, it’s unlikely any strong synergistic effects would be observed for antibiotics that are not effective and with specific modes of action would be seen. Rather AgNPs could for example facilitate the transport of hydrophilic antibiotics to the cell surface where the combination of direct action by the AgNPs and antibiotic inhibit cell wall synthesis leading to an increase in permeability allowing antibiotics to enter the cells more easily. While the mechanism(s) of the enhanced bactericidal effects of combined nanoparticles and antibiotics remains to be fully elucidated, there is some evidence to suggest differences in the size and shape of the prepared AgNPs and the bonding reaction between them enables the nanoparticle-antibiotic mixture to better interact with the pathogen (Pal et al., 2007; Panacek et al., 2016; Perveen et al., 2018).

Liu et al. (2010) showed that at our biocompatible concentration of 1.0 μg/mL, the toxicological activities of AgNPs in four human cell were size-dependent (Liu et al., 2010). The highest toxicity as demonstrated by changes in cell morphology and cell membrane damage, was seen with 5 nm particles which was higher than for Ag+ ions alone. Some toxicity was observed for 20 nm AgNPs, while no damage was seen with 50 nm nanoparticles indicating that the toxicity was negatively correlated with the nanoparticle size. These results correlate with the findings of this study where a nanoparticle size of ∼26 nm could be expected to show some mild level of toxicity over time. Similar results correlating toxicity with small nanoparticle size has also been observed for other nanoparticles such as gold (Pan et al., 2007).

The broth microdilution and disk diffusion experiments clearly demonstrated that 1 μg/mL of AgNPs alone had no antibacterial effects. However, when combined with 11 common antibiotics at the CLSI recommended MIC, enhanced antibacterial effects were observed against multiple bacterial species. The AgNPs and antibiotic combination was more effective against the gram-positive bacterial species tested although some reported studies have observed more effectiveness on gram-negative bacterial species (Fayaz et al., 2010; Perveen et al., 2018). This may be due to different species tested or properties of the AgNPs including their size. The precise mechanism for the enhancement of the synergistic antibacterial effects have not been fully delineated. It’s unlikely that structural differences in the bacterial cell wall between gram-positive and gram-negative bacterial species. is likely to completely explain the results of the study but could be a potential reason as the antibiotics with cell wall lysis capability, e.g., Amp10 along with AgNPs can cause serious damage to the bacterial cells (Fayaz et al., 2010). Metal depletion of the outer membrane in gram-negative bacteria has been suggested as a possible mechanism (Amro et al., 2000) and this is facilitated by the demonstrated negative zeta potential of the AgNPs in this study. In gram-positive bacteria, chelation of the nano-silver with available hydroxyl and amido groups on the antibiotics prevents DNA from unwinding resulting in serious damage downstream (Batarseh, 2004). While bacterial resistance to silver ions has been established, the appeal of using AgNPs was initially buoyed by the lack of reports of resistant bacterial species. Resistance to AgNPs by two gram-negative bacterial species (*E. coli*, and *P. aeruginosa)* after repeated exposure has now been reported (Panacek et al., 2018). Whether this resistance would be maintained with a combined AgNPs and antibiotic regimen, however, is yet to be determined.

## Conclusion

AgNPs used at concentrations shown to be biocompatible can synergistically increase the antibacterial effectiveness of antibiotics against multiple bacterial species tested. Moreover, antibiotic resistance shown by some bacterial species tested could be overcome by the addition of AgNPs, thus broadening the overall antibacterial potential. Further *in vivo* antibacterial studies using appropriate animal infection models treated with AgNPs alone or combined with specific antibiotics are required to confirm the potential therapeutic use of this novel strategy to tackle emerging microbial infections.

## Data Availability Statement

All datasets generated for this study are included in the article/Supplementary Material.

## Author Contributions

DI designed and performed all the cell biology, microbial work, and including manuscript drafting. DI and PK designed nanoparticles work. PK generated nanoparticles and helped in characterization. RL provided advice and was involved in designing the work, and made input in manuscript. SH was involved in designing experiment, manuscript drafting.

## Conflict of Interest

The authors declare that the research was conducted in the absence of any commercial or financial relationships that could be construed as a potential conflict of interest.

## Supplementary Material

FIGURE S1 **(A)** Gram-positive bacterial species tested: Antibacterial activity of AgNPs alone (1, 2, 5 μg, *c* = 0 μg); antibiotic alone (G +ve ABX, G −ve ABX) and synergistic antibacterial potential of 1 μg AgNPs combined with each of the 11 antibiotics tested. Lower case letters denote the antibiotic tested as described in Table 1. **(B)** Gram-negative bacterial species tested: Antibacterial activity of AgNPs alone (1, 2, 5 μg, *c* = 0 μg); antibiotic alone (G +ve ABX, G −ve ABX) and synergistic antibacterial potential of 1 μg AgNPs combined with each of the 11 antibiotics tested. Lower case letters denote the antibiotic tested as described in Table 1.
