# Supplementary figures and images for: Silver Nanoparticles at Biocompatible Dosage Synergistically Increases Bacterial Susceptibility to Antibiotics
Source: Front Microbiol. 2020 May 27;11:1074. doi: 10.3389/fmicb.2020.01074 (PMC7326045; doi:10.3389/fmicb.2020.01074)

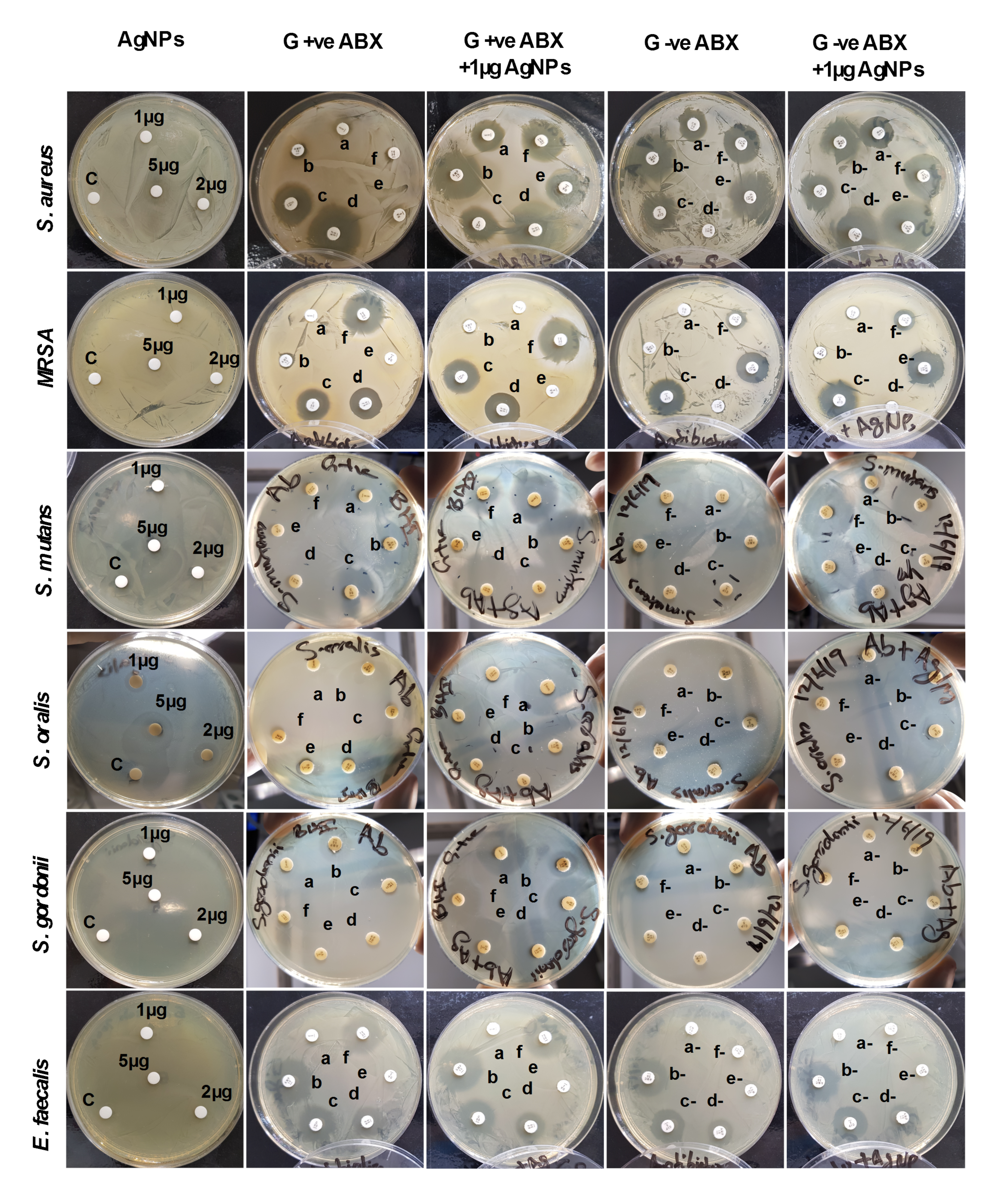

Supplement: FIGURE S1 — (A) Gram-positive bacterial species tested: Antibacterial activity of AgNPs alone (1, 2, 5 μg, c = 0 μg); antibiotic alone (G +ve ABX, G −ve ABX) and synergistic antibacterial potential of 1 μg AgNPs combined with each of the 11 antibiotics tested. Lower case letters denote the antibiotic tested as described in Table 1. (B) Gram-negative bacterial species tested: Antibacterial activity of AgNPs alone (1, 2, 5 μg, c = 0 μg); antibiotic alone (G +ve ABX, G −ve ABX) and synergistic antibacterial potential of 1 μg AgNPs combined with each of the 11 antibiotics tested. Lower case letters denote the antibiotic tested as described in Table 1. [file Data_Sheet_1.zip › Figure S1 A.tif]

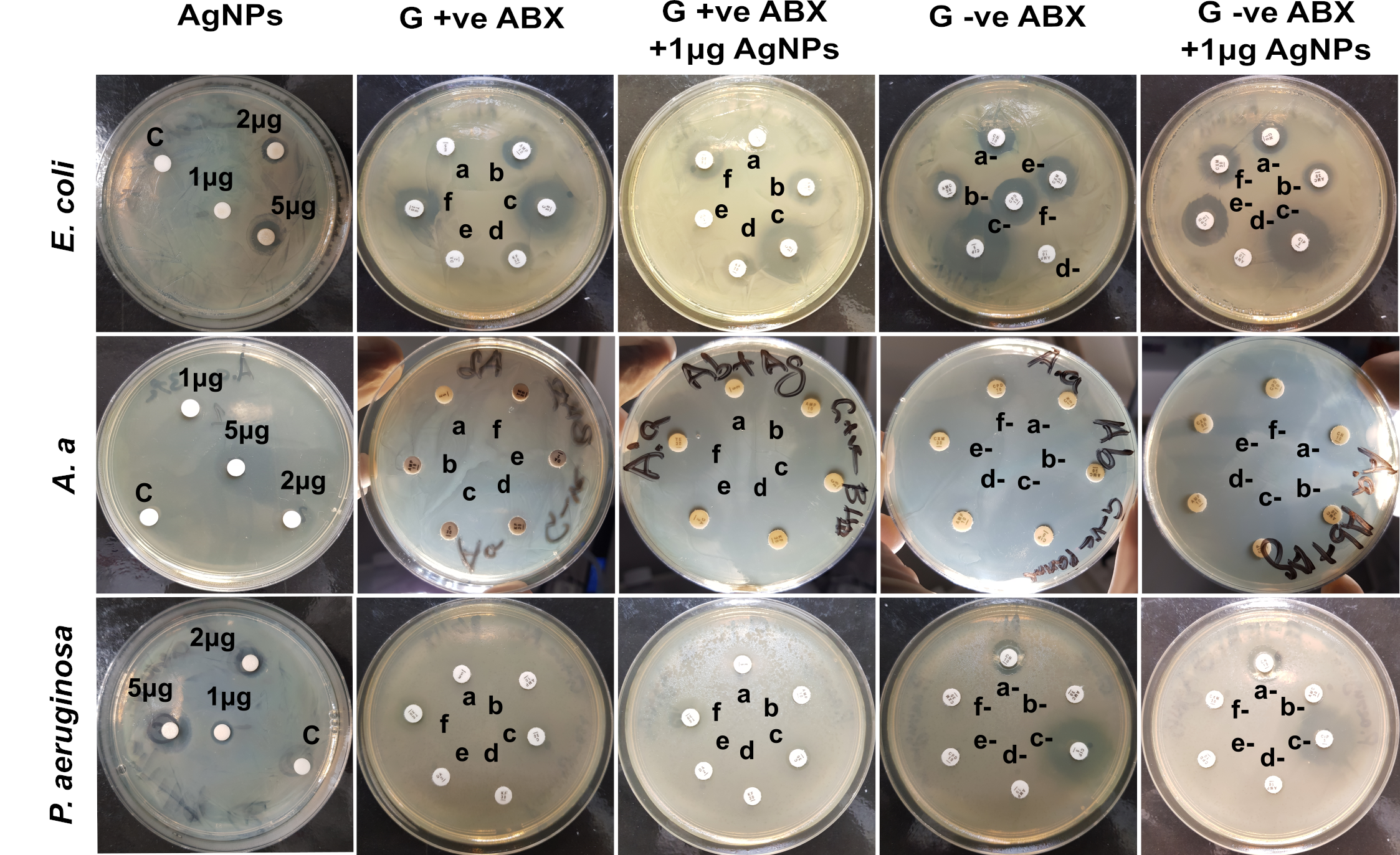

Supplement: FIGURE S1 — (A) Gram-positive bacterial species tested: Antibacterial activity of AgNPs alone (1, 2, 5 μg, c = 0 μg); antibiotic alone (G +ve ABX, G −ve ABX) and synergistic antibacterial potential of 1 μg AgNPs combined with each of the 11 antibiotics tested. Lower case letters denote the antibiotic tested as described in Table 1. (B) Gram-negative bacterial species tested: Antibacterial activity of AgNPs alone (1, 2, 5 μg, c = 0 μg); antibiotic alone (G +ve ABX, G −ve ABX) and synergistic antibacterial potential of 1 μg AgNPs combined with each of the 11 antibiotics tested. Lower case letters denote the antibiotic tested as described in Table 1. [file Data_Sheet_1.zip › Figure S1 B.tif]
